# Microstructure Study and Linear/Nonlinear Optical Performance of Bi-Embedded PVP/PVA Films for Optoelectronic and Optical Cut-Off Applications

**DOI:** 10.3390/polym14091741

**Published:** 2022-04-25

**Authors:** H. Elhosiny Ali, Mohammad Abdel-Aziz, Ashraf Mahmoud Ibrahiem, Mahmoud A. Sayed, Hisham S. M. Abd-Rabboh, Nasser S. Awwad, Hamed Algarni, Mohd. Shkir, M. Yasmin Khairy

**Affiliations:** 1 Advanced Functional Materials & Optoelectronic Laboratory (AFMOL), Department of Physics, Faculty of Science, King Khalid University, P.O. Box 9004, Abha 61413, Saudi Arabia; hibrahim@kku.edu.sa (H.E.A.); dr_ashraf9@yahoo.com (A.M.I.); frrag75@gmail.com (M.A.S.); halgarni@kku.edu.sa (H.A.); 2 Physics Department, Faculty of Science, Zagazig University, Zagazig 44519, Egypt; 3 Research Center for Advanced Materials Science (RCAMS), King Khalid University, P.O. Box 9004, Abha 61413, Saudi Arabia; 4 Department of Physics, Faculty of Science, Al-Azhar University, Cairo 11884, Egypt; m_aziz76@yahoo.com; 5 Physics Department, Faculty of Science, Aswan University, Aswan 81528, Egypt; 6 Physics Department, Faculty of Science, Al-Azhar University, Assiut 71524, Egypt; 7 Department of Chemistry, Faculty of Science, King Khalid University, P.O. Box 9004, Abha 61413, Saudi Arabia; habdrabboh@kku.edu.sa (H.S.M.A.-R.); aawwad@kku.edu.sa (N.S.A.); 8 Department of Chemistry, Faculty of Science, Ain Shams University, Cairo 11566, Egypt; 9 Department of Chemistry and University Centre for Research & Development, Chandigarh University, Mohali 140413, Punjab, India; 10 School of Science and Technology, Glocal University, Saharanpur 247001, Uttar Pradesh, India

**Keywords:** PVA/PVP, XRD/FTIR, SEM, optical absorption parameters, optical transition bandgap ($E_{\mathrm{gi}}^{\mathrm{opt}}$), optical limiting characteristic (OLC), L/NL optical parameters

## Abstract

Hybrid polymer films of polyvinyl pyrrolidone (PVP)/polyvinyl alcohol (PVA) embedded with gradient levels of Bi-powder were prepared using a conventional solution casting process. XRD, FTIR, and SEM techniques have been used to examine the micro/molecular structure and morphology of the synthesized flexible films. The intensities of the diffraction peaks and transmission spectrum of the PVP/PVA gradually declined with the introduction of Bi-metal. In addition, filler changes the microstructure surface of the pure film. The modification in the microstructure leads to an enhancement in the optical absorption characteristic of the blend films. The indirect allowed transition energy was calculated via Tauc’s and ASF (Absorption Spectra Fitting) models. The decrease in the hybrid film’s bandgap returns to the localized states in the forbidden region, which led the present films to be suitable for photo-electric, solar cell, etc., applications. The relation between the transition energy and the refractive index was studied. The enhancement in the refractive index with Bi-metal concentrations led to use the as-prepared films in optical sensors. The rise of Bi-metal concentrations leads also to the improvement of the nonlinear susceptibility and refractive parameters. The optical limiting characteristics revealed that the higher concentration dopant films reduce the light transmission intensity which is appropriate for laser attenuation and optical limiting in photonic devices. The results suggest that hybrid films are promising materials in a wide range of opto-electronic applications.

## 1. Introduction

Polymer materials have fascinated scientists because they are safe, economical, plentiful, and have eco-sustainable properties and extensive application in technical and scientific study. They can be used in optoelectronics, solar cells, UV-filters, coatings, photovoltaics, light-emitting diodes (LEDs), laser production, as well as several other potential applications [1,2,3,4,5]. The synthesis and examination of various polymer blends recently had a tremendous degree of concern. A polymer blend is a substance that combines two or sometimes more polymers to improve the product’s behavior with appropriate properties. It may have a distinct feature, enhancing the advantages of both additive polymers and improving their desirable characteristics. Moreover, they are dependent on the miscibility degree of the host polymers [6,7,8,9]. Their properties can be modified by adding different substances and particles [3,4,5]. Therefore, composites can be produced because the polymeric materials create a miscible solvent of strong hydrogen bonding between the molecules of the constituents [10].

Poly(vinyl alcohol) (PVA) [(C_2_H_4_O)_n_] is an exciting polymer due to its physical, chemical, mechanical, and thermal characteristics. Moreover, PVA has particular features such as semi-crystalline, adhesive properties, and water-soluble. It is suitable for a wide range of scientific, biomedical, and technological applications [11,12,13,14]. However, another polymer material such as poly (vinyl pyrrolidone) (PVP) [(C_6_H_9_ON)_n_] show biological compatibility, an amorphous structure, soft processing capability, good environmental stability, and outstanding solubility, which make it suitable for a variety of applications such as optics and photonics [2,6].

PVP and PVA are considered the famous and desired polymers as perfect and operative binders in the production of optical responsive materials employed in the designing of sensor systems, optoelectronics, and organic electronic systems [12]. They are highly compatible thermodynamically. Their blend interrelates through hydrogen bonding between the PVA’s hydroxyl (−OH) and the PVP’s carbonyl (C=O) groups. PVA and PVP polymer solutions are the most widely used, economical, and easily accessible methods for creating novel materials with advanced properties for promising optoelectronic applications [13]. The blend polymers with inorganic nano-dopants, metal salts, rare-earth ions, ceramic, or other additives, can be an appropriate composite for several technological and industrial fields [7]. Badawi et al. [2] examined the effect of tin sulfide (SnS) on the PVP/ PVA blend’s physical properties. The concentration of SnS dopant influences the structural and optical properties of the synthesized polymer composite films. However, Ali et al. [13] investigated the optical properties of PVP/ PVA polymeric films loaded with lanthanum ions (La^3+^). The participation of the La^3+^-ions results in substantial changes in such properties. PVP/ PVA: silver sulfide (Ag_2_S) composites, as described by Aziz et al. [14], have widespread applications in optoelectronic and electronic devices, including photoconductive cells, solar cells, and photodetectors.

Various polymer-metal composites were prepared using various techniques, and their properties were investigated by several research groups [15,16,17,18]. Most of the previous work concerns the study of metal oxide’s effect on the microstructure, mechanical, dielectric, linear optical properties of blend composites [2,7,14,18]. Here, the influence of Bi-metal percentage on the microstructural and morphology of PVA/PVP blend matrix was detailly investigated by X-ray diffraction (XRD), Fourier transform infrared (FTIR), and scanning electron microscope (SEM). Then, the physio-chemical relation was observed by studying the band gap, Urbach energy, absorption edge, refractive index—energy gap relation, and nonlinear optical parameters for solar cell, sensors, and photoelectrical applications. Moreover, for laser attenuation in photonic devices, the optical limiting of the films was studied as a function of Bi-metal concentrations.

## 2. Experimental Details

### 2.1. Materials

Polyvinyl alcohol PVA [(C_2_H_4_O)_n_] with molecular weight (M.W. = 115,000 g/mol) and polyvinyl pyrrolidone PVP [(C_6_H_9_ON)_n_] with M.W. = 40,000 g/mol were sourced from Alfa aesar, Germany. Both have *4N* purity and were used in powder form without extra purification. Bi-metal powder with *3N* purity and molecular weight (M.W. = 208.98 g/mol) was used as a dopant substance. It was received from LOBA Chemie.

### 2.2. Pure and Hybrid Films Preparation

Pristine PVA, PVA/PVP polymer blend, and composite polymer blend films with Bi-metal powder concentrations of 0.001, 0.01, 0.05, and 0.1 g were prepared using the conventional solution-casting method. First, each polymer’s solution was fabricated separately by dissolving 45 g from PVP and PVP powders within 1 L of double distilled water (DDW). Next, the 50%PVA/50%PVP mixtures were stirred at 90 °C for homogeneous, transparent, and viscous solutions. After that, the blend solution was mixed with different weights of Bi-metal and ultrasonically homogenous distributed with 60 mL of the blend for 2 min. Finally, the weights of the polymer blend *w_b_* and the Bi-dopant *w_Bi_* were used to calculate Bi-metal’s dopant concentrations (*x* wt.%).

(1) $$w_{Bi}\left(\%\right)=\frac{w_{Bi}}{w_{Bi}+w_{b}}\times 100$$

The homogenous and final solutions were then poured on 80 mm Petri glass dishes. The solutions were dried in a warmer furnace at 35 °C for three days to create fully dried homogeneous and bubble-free polymer films. The produced polymer samples were removed from the glass dishes and cut into 2 × 2 cm^2^ pieces that could be used for all measurements. The final films have thickness with a 0.40 mm average. These films were labeled: pristine PVA, PVA/PVP blend, PVA/PVP: 0.037 wt.% Bi (1Bi-blend), PVA/PVP: 0.37 wt.% Bi (2Bi-blend), PVA/PVP: 1.8 wt.% Bi (3Bi-blend), and PVA/PVP: 3.7 wt.% Bi (4Bi-blend).

### 2.3. Experimental Techniques

Pieces of regular thickness polymer films were carefully placed on a specimen holder of Shimadzu diffractometer (XRD-6000) (Kyot, Japan)-copper target (*λ_Cu-kα_* = 1.54108 Å). It was working at a *V* = 40 kV and *I* = 25 mA. The samples’ examination was done in a range of 10° ≤ 2θ ≤ 70° and rate 0.02/s to distinguish all phases formed in the polymer films. 6700 FTIR spectrometer was operated to evaluate the sample’s transmission spectra with a resolution 4 cm^−1^. The synthesized polymer film’s surface morphology was analyzed using a scanning electron microscope (SEM) (JSM-6360 model) (Peabody, MA, USA) at 10 kV operating voltage.

A double beam spectrophotometer (V-570 model—JASCO) (Easton, MD, USA) was used to measure the pure and composite blend film’s optical transmission and absorbance characteristics in the wavelength range of 200–1000 nm.

For optical limiting measurement, a sensitive laser power meter (Lab-Master Ultima, COHERENT, Santa Clara, CA, USA) and lens of focal length 10 cm were set to detect the output beam and focus the laser beam. In addition, He−Ne and green lasers with constant energy (0.5 mW and 15.4 W) and wavelengths 632.8 and 533 nm, respectively, were operated to show the influence of Bi content on the film’s absorption.

## 3. Results and Discussion

### 3.1. X-ray Diffraction (XRD) Investigation

**Figure 1:**
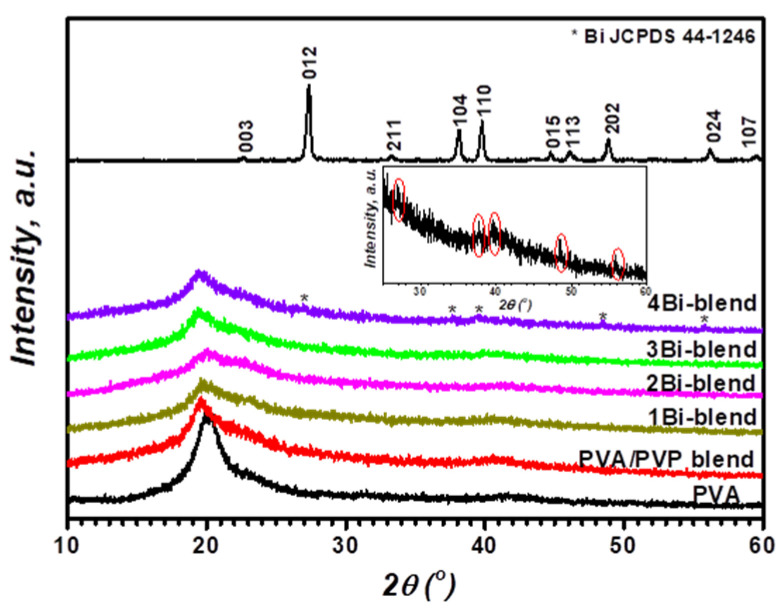
XRD patterns of pristine PVA, PVP/PVA blend, Bi-metal powder, and Bi-blend hybrid samples.

The XRD pattern of pristine and Bi-blend composites can be seen in Figure 1. The pattern of the PVA demonstrates two diffraction peaks at approximately 2θ = 20° and 41.7°. This is attributed to the semi-crystalline feature of the pristine PVA [9]. Due to the −OH groups within the main matrix of PVA, it involves good intermolecular and intramolecular hydrogen bonding [19]. However, the blend film’s pattern shows that the intensity of the main crystalline peak of the PVA was considerably shifted to 19.86°, and the broadest of the bandwidth. This means the amorphous portion in the mixed sample is more significant than that in the pristine PVA sample. It is known that PVP has an amorphous structure, which is an appropriate polymer for various applications [20]. Therefore, the mixture between two polymers causes a reduction in PVA crystallinity and an amorphous increment in the blend matrix. This demonstrates the better miscibility and connectivity between the −OH groups of PVA and C=O of PVP groups [16]. Moreover, this decline becomes more significant with Bi-metal content due to the disruption of particles in the crystalline portion of the blend matrix, making the amorphous performance major in the composite/hybrid films. Therefore, there is a direct correlation between the crystallinity degree and the peak intensity, as recognized via El-Naggar and coauthors [21]. They noticed that the intensity of PVA/PVP diffraction decreased with the increase in amorphous nature by adding filler. The XRD pattern of Bi-nanoparticle shows reflection peaks corresponding to the rhombohedral structure with space group R3m (#166). This diffraction was compatible with the file JCPDS: 44–1246 for pure Bi-metal [22,23]. The addition of a low ratio of Bi-particles to the blend presented no specific peak relating to the crystal structure of the powder. However, the composite’s crystallinity decrease suggests a complexation of Bi particles in the matrix chains via the hydroxyl and carbonyl groups. By increasing the ratio of Bi-metal powder to 3.7 wt.%, small peaks were found at 2θ = 27.32°, 38.06°, 39.70°, 48.84°, 56.18°, 62.32°, and 64.5° related to the reflections from the crystallographic Bi-powder. The presence of specifically distinguished peaks is due to the Bi-metal powder accumulation on the polymer matrix at a high concentration. It was reported that the filler’s concentration affected the crystallinity of the polymer material [24,25]. The XRD of PVA doped with metal shows a decrease in the intensity of diffraction peaks of PVA [26]. This is related to the growth of distortion and imperfection due to the strong interaction between the PVA matrix and the metal. Herein, the peaks observed from the Bi-metal increased with the concentration of bismuth in polymer, and the intensity of diffraction peaks from PVA/PVP decreased, probably due to interaction between the Bi-particles and the hydrogen bond. These observations are consistent with other prior research on PVA filled by CdSe quantum dots and PVP/PVA doped with Pb(NO_3_)_2_ [27,28].

**Figure 2:**
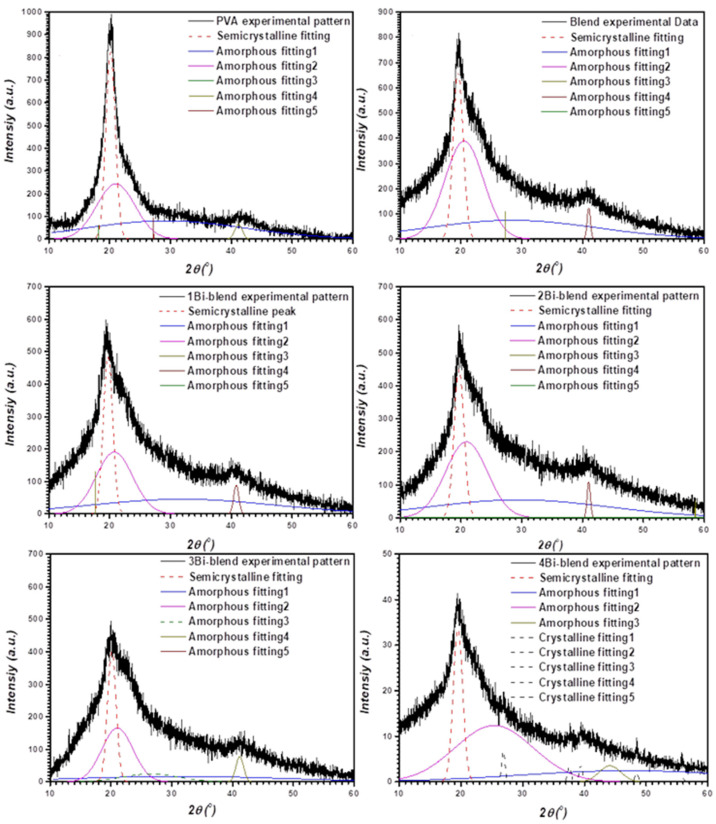
Deconvoluted XRD patterns of pure and composite films.

The degree of crystallinity of the pure and composite films was estimated by fitting their diffraction patterns of the main peak via Fityk 0.8.9 software (Figure 2). The crystalline fraction (*X*_*cryst*._) was calculated using the next relation [29].

(2) $$X_{cryst.}=\frac{A_{cryst.}}{A_{\left(cryst.+amorph.\right)}}\;\%$$

*A_cryst._* and *A*_(*cryst.* + *amoph.*)_ represent the area under the crystalline and all curves. The values are reported in Table 1. A significant decrease in crystallinity was observed in Bi-blend composite films. However, for 4Bi-blend composite film, the crystallinity increased again due to the presence of a high ratio of Bi-crystalline particles. This behavior was reported in various studies of doping polymeric material with different fillers [30,31,32]. The intensity of diffraction peaks of PVA decreased with increasing cobalt metal concentration, probably due to interaction between the Co particles and the hydrogen bond [29]. This is related to the growth of imperfection and distortion due to the strong interaction between the PVA matrix and the metal. Herein, the peaks observed from the Bi-metal increased with the increasing concentration of bismuth in the polymer, and the intensity of diffraction peaks from PVA/PVP decreased.

### 3.2. FTIR Investigations

**Figure 3:**
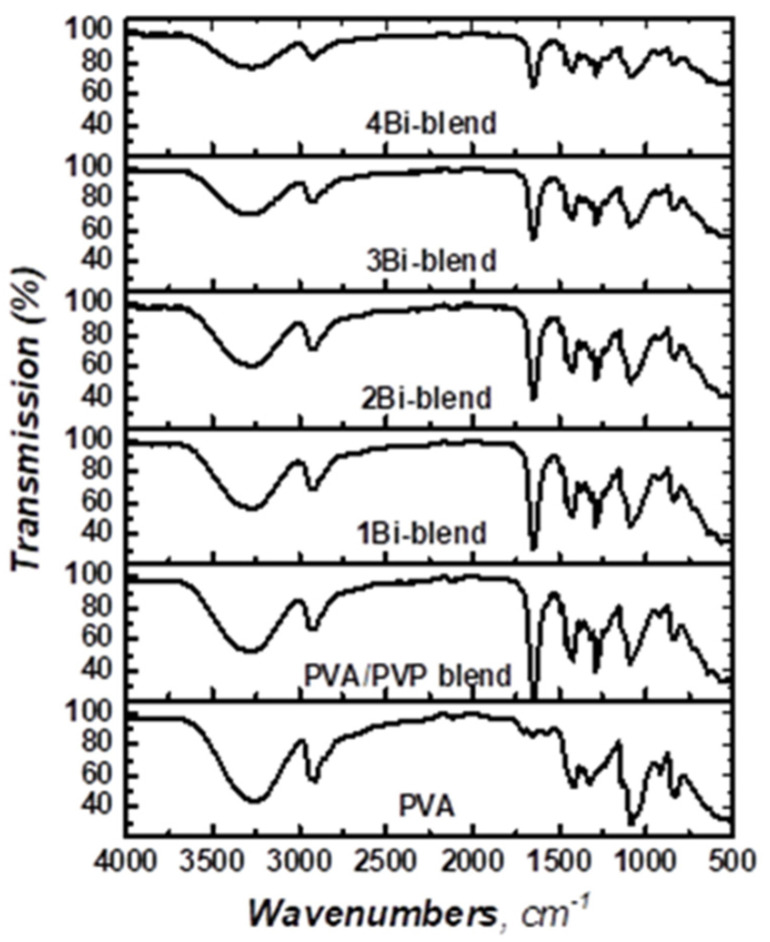
FTIR spectra of pristine PVA, PVP/PVA blend, and Bi-blend hybrid films.

FTIR spectroscopy is an effective widespread technique applied to acquire a broad range of infrared spectra that describes and identifies the interactions of polymer matrix with dopant materials [33]. Figure 3 illustrates FTIR transmission spectra over the range 4000–500 cm^−1^ of pristine PVA, PVP/PVA blend, and Bi-blend composites. The vibration bands of PVA were centered at 3258, 2922, 1416, 1323, 1085, 917, and 839 cm^−1^ for −OH, −CH, −CH_2_ bending, −CH_2_ waging, C–O, C−C, and −CH stretching vibrations, respectively [8,10]. Moreover, the peaks located at 1647, 1375, and 1290 cm^−1^ match with C=O stretching, −CH_2_ bending, and CH_2_ twisting or wagging vibrational modes of the PVP chains, respectively [34,35]. Moreover, the band centered at 1495 cm^−1^ corresponds to the characteristic vibration of C=N (pyridine ring) of PVP [36]. The intensity of such peaks slightly decreased with the increase in Bi-metal powder concentration in the blend relative to the pure films. This is similar to the result of the XRD study. Thus, it can be deduced that the Bi-particles interact with the backbone chains of the hydroxyl groups in PVA and carbonyl (C=O) groups in PVP [37]. PVA/PVP matrix feature dominates at a small Bi-metal percentage, while the decrease in the intensity of the functional vibration groups in the matrix reflects the strong interactions between the metal and the PVA/PVP matrix.

### 3.3. Morphological Analysis

**Figure 4:**
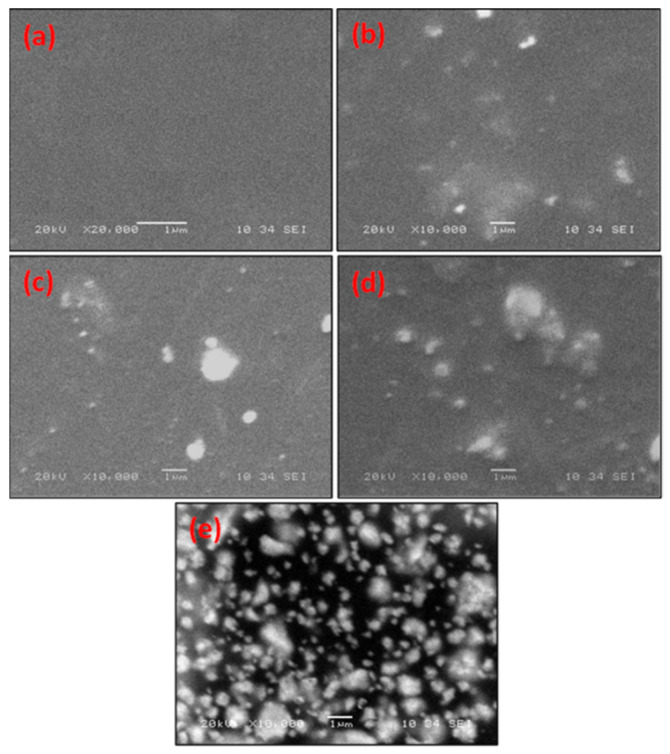
SEM micrographs of the synthesized polymer (**a**) PVA/PVP, (**b**) 1Bi-blend, (**c**) 2Bi-blend, (**d**) 3Bi-blend, and (**e**) 4Bi-blend.

The surface morphology of the synthesized pure and hybrid films were investigated using SEM. Pure and different Bi-contents loaded blend composite images are shown in Figure 4a–d. The blend film has a homogeneous and smooth surface without cracks (Figure 4a). These results are consistent with PVA/PVP blend film [2,12,32]. The bright area on the surface of the composites is related to the Bi-particles that are randomly distributed over the polymer blend surface. This induces a significant change in the surface morphology of the PVA−PVP matrix. There is little agglomeration when the dopant content increases to 3.7 wt.% [2Bi-blend] (Figure 4c). This agglomeration increased in 4Bi-blend film. These clusters are dispersed throughout the film’s surface, suggesting proper polymer–particle interaction; the organic–inorganic components in the polymer composites are compatible [38]. Thus, the hybrid film’s surface roughness increases with the increase in concentration of Bi-metal. Adding high percentage of Bi-metal causes the particles to agglomerate (as seen by SEM images) and more incident light to be absorbed or reflected in the UV–Vis spectra.

### 3.4. UV-Vis-NIR Spectroscopy

#### 3.4.1. Optical Properties

**Figure 5:**
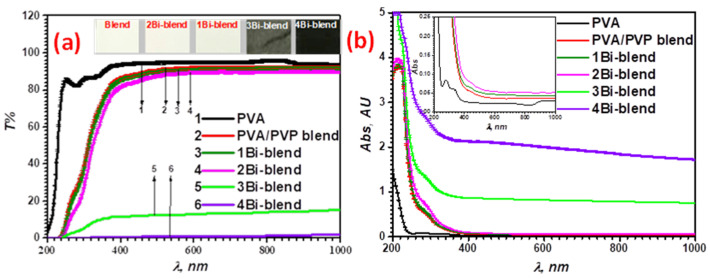
UV–Vis-NIR spectra of pristine PVA, PVP/PVA blend, and Bi-blend hybrid films (**a**) transmittance and (**b**) absorbance.

The analysis of optical characteristics of the materials is a valuable method for investigating the band structure as well as the density of electronic states [36]. The optical features of polymer blends often change when mixed with a filler [32]. The regular optical measurement of pristine and Bi-blend hybrid samples was analyzed in the spectral range 200–1000 nm, as illustrated in Figure 5a,b. Figure 5a shows the highest transmission for pristine PVA film (*T*%~95%). This optical transmission decreases to about 93.5% for the pristine blend. This results from the mixture through inter-chain hydrogen bonding between PVA hydroxyl groups and PVP carbonyl groups. Therefore, the Bi-metal affects the optical features of the blend matrix. The transmittance drops significantly in the UV-visible region with the increase in Bi-metal powder to 3.7 wt.% in the PVA/PVP blend due to the agglomeration of the particles at the surface. Thus, the incident light is absorbed or dispersed and leads to a decrease in transmissions significantly [21,39]. The feature can be considered a novel implementation for UV block and laser attenuation. In the pristine PVA, tiny peaks at about 279 nm and 333 nm were noticed, indicating the presence of an electronic movement from *n* to π* and *π* to π* electronic transitions [40]. However, this peak is wholly disappeared in the pristine blend and Bi-blend samples. This result is well matched with those of PVA/PVP filled with SnS nanocomposites [2].

Regarding the optical absorption spectra in Figure 5b, the high absorption level was improved by the 4Bi-blend film. The absorption edge of pristine and Bi-blend composites moves to lower energy (higher wavelength) than pristine PVA film. Therefore, the bandgap changes with Bi-metal in the blend, suggesting the complex interaction. This confirms the creation of levels between VB and CB, leading to easy electron transfer throughout the structure [41].

One of the significant parameters for investigating the variation of the polymer material’s band structure is the absorption coefficient [42]. It offers valuable information about the nature of the energy of the forbidden optical gap, which is exploited in all future applications. The absorption coefficient (*α*) can be calculated from the absorption *A*(*λ*) and the thickness of the synthesized films, *d*, by applying the Beer–Lambert law [43]:

(3) $$\alpha =\frac{2.303\times A\left(\lambda \right)}{d}$$

**Figure 6:**
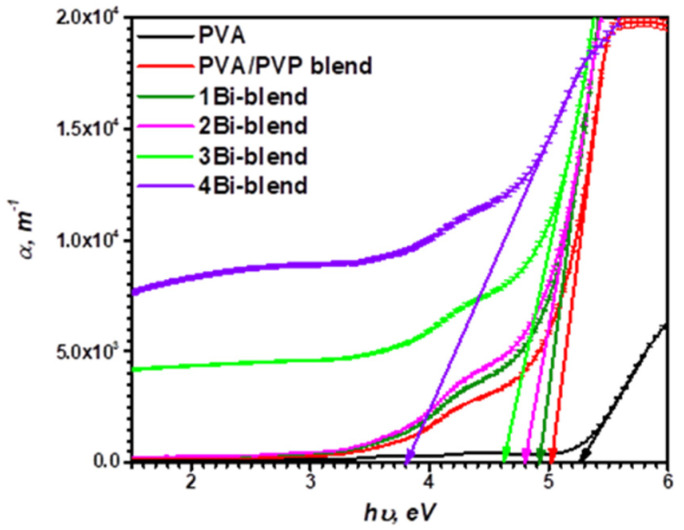
Variation of the coefficient of optical absorption (*α*) via the photon energy (*hν*) of pristine PVA, PVP/PVA blend, and Bi-blend hybrid films.

The variations of the *α*-spectra with the photon energy, *hυ*, are shown in Figure 6. The *α* values for all films are evaluated within the range of 10^4^ m^−1^. This suggests that the energy required is sufficient to excite the electrons from LUMO (lowest unoccupied molecular orbital) to HOMO (highest occupied molecular orbital) [44]. By extrapolating the sharp part of the absorption graph to intersect the *hυ* axis at the point of *α* = 0, the absorption edge’s energy (*E_e_*) could be calculated. Table 1 presents the values of the measured *E_e_* of the polymer samples. It is decreased from 5.25 eV of PVA to 5.01 eV for the blend after combining with PVP representing the decrease in the optical gap of PVA [45]. This confirms the significant interactions between PVA and PVP matrices. Moreover, the absorption edges move to the lower energy direction (3.8 eV) by loading the Bi-powder (4Bi-blend). This result matches with the XRD and FTIR performances of the composite films due to the influence of Bi-metal percentage on the microstructure of the PVA/PVP matrix. This value is close to those reported for PVA/PVP filled by Ce^3+^-ions [46].

#### 3.4.2. Urbach’s Tail Energy and Optical Energy Gap Calculations

**Figure 7:**
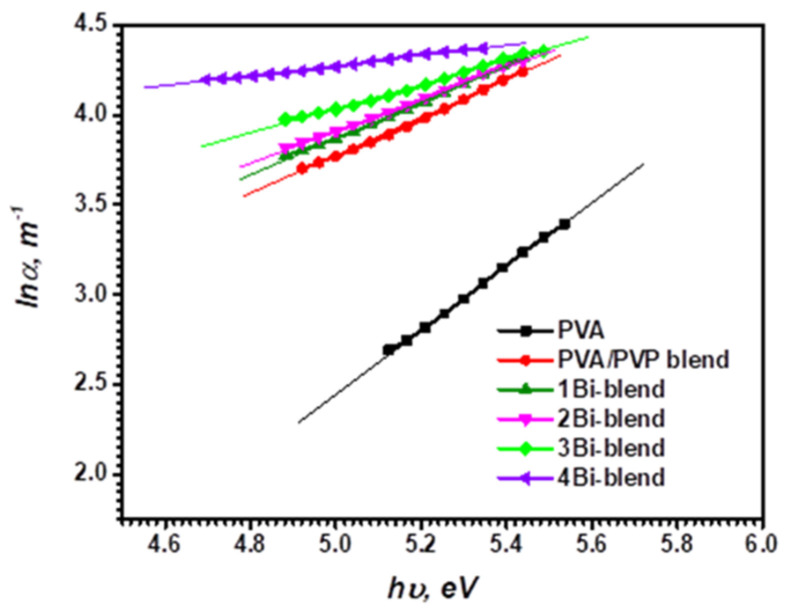
Variation of lnα versus the photon energy (*hν*) of pristine PVA, PVP/PVA blend, and Bi-blend hybrid films.

The performance of several solid-state devices (emissive displays, integrated optical circuits, optical sensors, etc.,) can be enhanced by applying a high refractive index coating onto the sensing surface of the device (a regular change from the high refractive index of the active circuitry to the low index of air permits light to be coupled more efficiently). The refractive index of the materials varied with the change in the energy gap which is affected by the localized states in the forbidden band. The change in the absorption coefficient was contributed to the band tail (Urbach’s energy). The tail energy (*E_u_*) value indicates the defects and the disorder in the polymer matrix. It is located inside the prohibited bandgap close to the valence and conduction band’s edges and describes the localized states’ width [39]. Urbach proposed that this band tail is defined through the following empirical relationship [17]:

(4) $$ln\alpha =ln\beta_{o}+\frac{h\upsilon }{E_{u}}$$

where *β_o_* is constant. Urbach’s tail energy (*E_u_*) was calculated by plotting the logarithm values of the absorption coefficient (*lnα*) versus the photon energy (*hυ*), as shown in Figure 7. By fitting the straight part of the graph, the inverse of its slope is the *E_u_* values. It varies between 0.973 and 3.007 eV for the Bi-embedded blend (Table 1). This value is higher than that of PVA/PVP filled with 10 wt.% MWCNTs [34]. The variations of *E_u_* with composition are related to the nature and the content of defects/disorders created in the prohibited gap. These results are compatible with the results obtained from the structure study.

The electron transitions of the materials typically depend on the incident photon’s energy, as suggested by Tauc’s law. The relationship between the *α* and the energy of the incident photon (*hυ*) was calculated in the region of strong absorption using the formula [47]:

(5) $${\left(\alpha E\right)}^{x}=K\left(E-E_{g}^{opt}\right)$$

**Figure 8:**
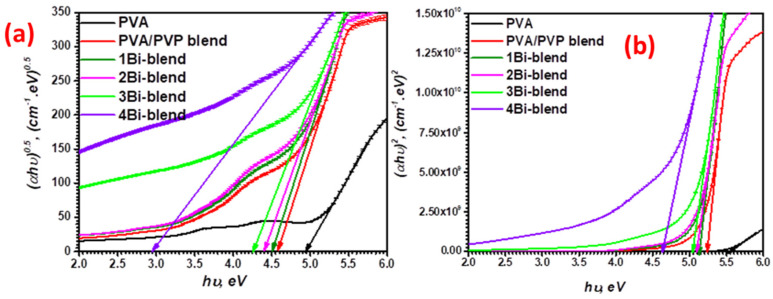
Indirect (**a**) and direct (**b**) optical energy band gaps of pristine PVA, PVP/PVA blend, and Bi-blend hybrid films.

*K* is the energy-independent band tail parameter and depends on the probability of electronic transition between the valance and conduction bands. *x* is the power parameter of transition type determined from the material’s nature, i.e., crystalline or amorphous. It can be 2, 1/2, 2/3, and 1/3 for direct permitted, indirect permitted, direct prohibited, and indirect prohibited transitions. The graphical relationship between (*αE*)*^x^* versus the photon energy (*E* = *hυ*) is presented in Figure 8. The distinct straight segment that corresponds to the material’s onset of absorption is observed in the graphs. By extrapolating this linear part to the *x*-axis (photon energy), each material’s band gap *E_opt_* can be specified.

Here, all synthesized polymer films have values less than 10^6^ m^−1^, describing the indirect electron transport. For estimating the optical bandgap, we plot (*αE*)^1/2^ and (*αE*)^2^ against photon energy (*hυ*), as shown in Figure 8. The results are reported in Table 1. The optical energy gap was reduced by raising the Bi-metal powder concentration to 3.7 wt.% due to the increment of localized states (trapping centers). These states are close to the valence and conduction bands, as they exponentially move into the prohibited band [48]. The result indicates that the PVA/PVP blend doped with Bi-metal is more effective than that reported for those doped with SnS_2_/Fe nanoparticles [49]. The present flexible polymers have effective optical performance.

**Table 1:** Absorption edge, Urbach energy, and optical bandgap of Bi-blend hybrid films.

Films	X_c_ (%)	*E_e_* (eV)	*E_u_* (eV)	$E_{gi}^{opt}\;(\mathrm{eV})\;\pm \;0.01$ (Tauc’s Relation)	$E_{gd}^{opt}\;(\mathrm{eV})\;\pm \;0.001$ (Tauc’s Relation)	$E_{gi}^{opt}\;(\mathrm{eV})\;\pm \;0.01$ (ASF Method)
PVA	26.45	5.254	0.566	4.935	5.539	5.022
PVA/PVP blend	25.67	5.018	0.936	4.573	5.238	4.600
1Bi-blend	23.80	4.925	0.973	4.496	5.143	4.526
2Bi-blend	19.86	4.804	1.073	4.406	5.095	4.439
3Bi-blend	19.42	4.617	1.301	4.257	5.041	4.290
4Bi-blend	23.20	3.800	3.007	2.938	4.623	3.031
Blend: 0–5 wt.% MWCNTs [34]	−	−	0.43–0.82	5.06–4.46	−	−
Blend: 0–10 wt.% SnS_2_/Fe [49]	−	−	−	5.09–3.85	5.26–4.49	5.20–4.18

The type of electronic transitions, whether direct or indirect, can be distinguished according to the absorption coefficient values (*α*). Electrons are supposed to be transported directly if *α* > 10^6^ m^−1^, and indirect for *α* < 10^6^ m^−1^ [50]. Thus, another approach depending on the fitting of the absorption spectra (*ASF*) model can be used to evaluate the optical energy bandgap. This model is thickness-independent according to the relationship [51]:

(6) $$A\left(\lambda \right)=D_{1}\lambda {\left[\frac{1}{\lambda }-\frac{1}{\lambda_{opt}}\right]}^{x-1}+D_{2}$$

(7) $$D_{1}=\left[d{\left(E\lambda \right)}^{x-1}/2.303\right]K$$

**Figure 9:**
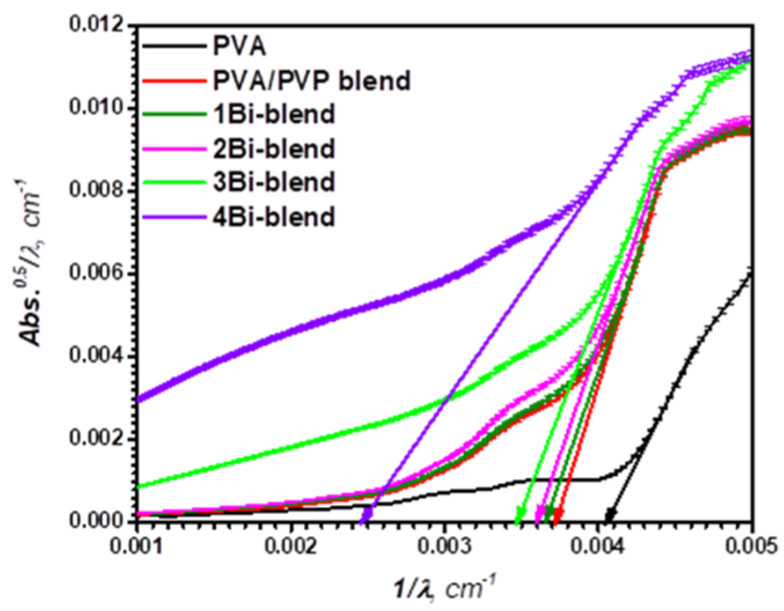
Variation of (Abs/λ)^1/2^ versus λ^−1^ of pristine PVA, PVP/PVA blend, and Bi-blend hybrid films.

The forbidden optical gap $E_{ASF}^{opt}$ (eV) was estimated by extrapolating the linear segment of (*Abs*^1/2^*/λ*) − 1/*λ* plot to (*Abs*^1/2^*/λ*)= 0, as shown in Figure 9. Then, the optical band gap was calculated by using the relationship: EASFopt=1240λopt. Finally, the $E_{ASF}^{opt}$ value of each film was recorded in Table 1. The bandgap calculated by the ASF approach is almost close to that from Tauc’s model.

**Figure 10:**
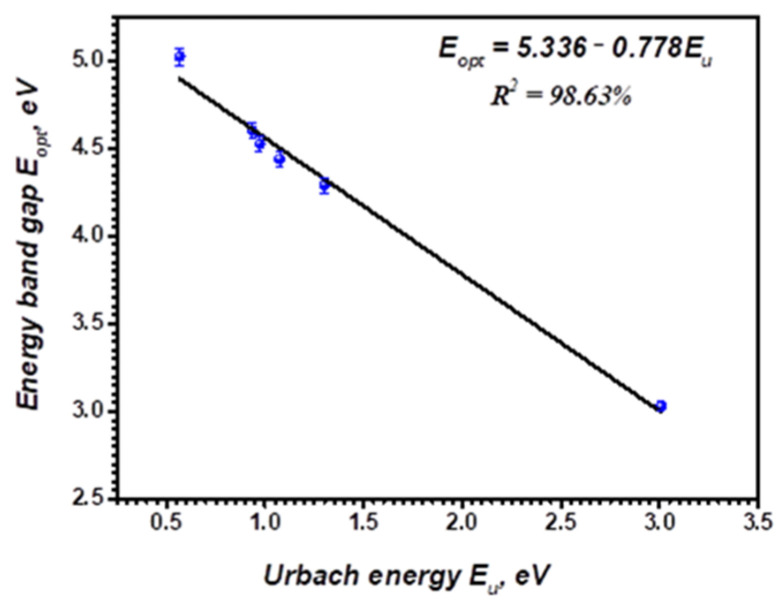
Variation of the average indirect optical band gap, $E_{opt}^{av}$, vs Urbach energy, $E_{u}$ , of pristine PVP/PVA blend, and Bi-blend hybrid films.

Figure 10 indicates a graphical relation between the indirect optical energy gap ($E_{ASF}^{opt}$) versus the values of Urbach energy $\left(E_{u}\right)$ for pristine and Bi-embedded blend. Thus, from these two approaches, we can estimate the following linear fitting formula:

(8) $$E_{ASF}^{opt}=5.336-0.778E_{u}$$

These observed results represent a typical attitude, since the bandgap reduction is due to localized states at the prohibited band’s boundaries. For many polymer films, this indirect attitude between *E_opt_* and the *E_u_* is indicated [52,53,54].

#### 3.4.3. Extinction Coefficient and Refractive Index Investigations

The coefficient of extinction index (*k*) is the quantity of energy absorbed when the electromagnetic radiation propagated through the material. It indicates the proportion of light losses owing to the penetrating material’s absorption and dispersion per unit distance. In addition, it depends on the structural defects and the amount of the material’s charged particles. The study of this parameter is essential for future optoelectronic applications of the materials under investigation. It is the imaginary component of the complex index of refraction, $\bar{n}=n-ik$, and evaluated in terms of *α* by using the relationship [55]:

(9) $$k=\frac{\alpha \lambda }{4\pi }$$

**Figure 11:**
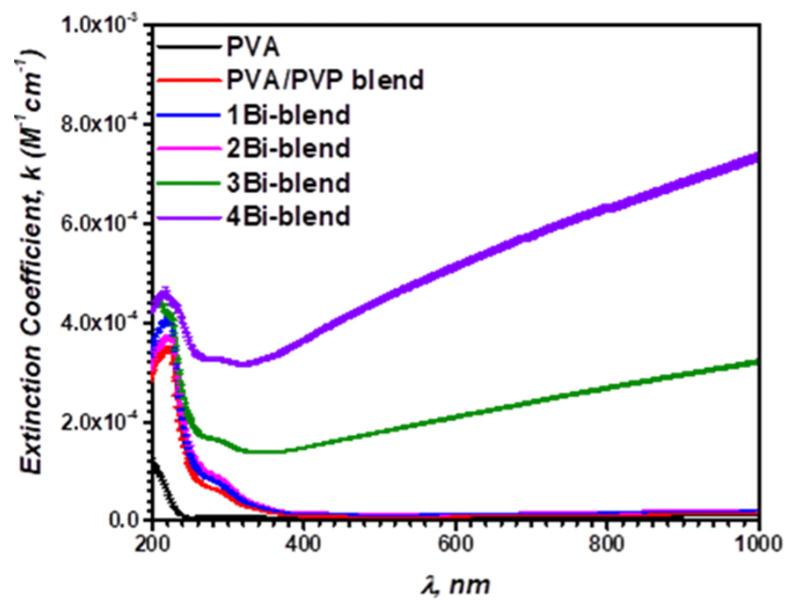
Variation of extinction (*k*) coefficient versus the photon wavelength (*λ*) of pristine PVP/PVA blend, and Bi-blend hybrid films.

The variation of *k* with the incident photon wavelength, λ, is shown in Figure 11. It is observed that *k*-values are substantially reduced as the wavelength increases between 200 and ~340 nm and then become constant in the visible region for the pristine PVA, blend, and blend films with lower Bi-dopant concentrations. Such low absorption index values are due to the high optical transmission for these polymer films within this zone (UV-visible). Furthermore, by increasing the Bi-dopant concentration to 3.7 wt.%, the *k*-values increased because the optical absorption for those composites is improved [56]. A similar behavior was observed for PVA/PVP blend doped with SnS_2_/Fe [49].

Moreover, the index of refraction, *n*, is an essential parameter for using the materials in the manufacturing of optical instruments, optoelectronic devices, optical switches, filters, light-emitting diodes, modulation, and waveguides [57,58]. It would be relevant to the ion electronic polarization and the local field within the optical substances [20]. Furthermore, there is a relation between their values and the energy gap, *E_opt_*. According to their significance in studying material’s band structures, these two essential parameters were studied intensively. The energy gap is commonly evaluated by the electromagnetic wave threshold absorption, while the transparency of the material is estimated utilizing the refractive index. The *n* value was calculated depending on the optical bandgap as [59]:

(10) n2−1n2+2=1−EASFopt20

Table 2 summarizes the calculated refractive index of each model. The variation of the *n* is related to the sample’s structural characterization (i.e., the optical bandgap). The obtained values are higher than PVA/PVP with SnS [2], Ag_2_S [17], and SnS_2_Fe [49].

**Figure 12:**
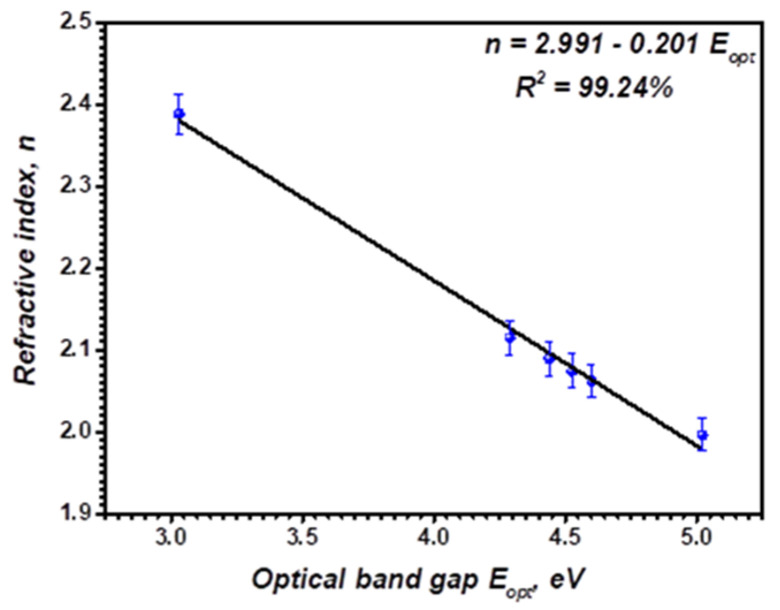
Variation of refractive index, *n* optical energy gap, $E_{ASF}^{opt}$, of pristine PVA, PVP/PVA blend, and Bi-blend hybrid films.

Figure 12 demonstrates the variation of *n* with the bandgap, $E_{ASF}^{opt}$, for pristine and Bi-blend hybrid films. By fitting the straight line, we can obtain a linear formula for the as-prepared polymer films as follows:

(11) $$n=2.991-0.201E_{ASF}^{opt}$$

The negative slope indicates that the refractive index behaves inversely with the energy gap value.

The high (*ε*_∞_) and static-frequency (*ε_o_*) dielectric constants are effective for various electronic instruments. They are calculated based on the formulas [54]:

(12) $$n=\sqrt{\varepsilon_{\infty }}$$

(13) $$\varepsilon_{o}=-33.26876+78.61805E_{opt}-45.70795E_{opt}^{2}+8.32449E_{opt}^{3}$$

Their values changed with the refractive indices and the optical energy gap, respectively, as reported in Table 2. The result indicates the linear optical parameters changed with the modification of the electronic structure of the blend by the Bi-metal content.

#### 3.4.4. Nonlinear Optical Parameters

Nonlinear optics play a major role in many of the optical applications such as optical signal processing, optical computers, ultrafast switches, ultra-short pulsed lasers, sensors, laser amplifiers, and many others. So, the calculation of nonlinear optical susceptibility and refractive index is important as present in the present work. The characteristics of nonlinear optical polymeric materials primarily depend on dopant concentration and host polymer properties. High-value nonlinear optical materials are commonly required to design various optoelectronic instruments [60]. Nonlinear material behavior is induced by large radiation intensities, such as lasers [61]. This is due to the induced polarization (*P*) and the applied electric field (*E*). Therefore, the nonlinear refractive index (*n*_2_), first-order susceptibility ($\chi^{\left(1\right)}$), and higher-order susceptibility ($\chi^{\left(3\right)}$) of the as-prepared polymer films must be examined. First, $\chi^{\left(3\right)}$ can be determined using the following relation:

(14) $$\chi^{\left(3\right)}=C{\left(\chi^{\left(1\right)}\right)}^{4}$$

where *C* = 1.7 × 10^−10^ esu, and $\chi^{\left(1\right)}$ is determined by the known linear refractive index (*n_av_*) using the formula:

(15) $$\chi^{\left(1\right)}=\frac{n^{2}-1}{4\pi }$$

Therefore, it is possible to estimate the *n_2_* value from the below relation:

(16) $$n_{2}=\frac{12\pi \chi^{\left(3\right)}}{n_{av}}$$

**Figure 13:**
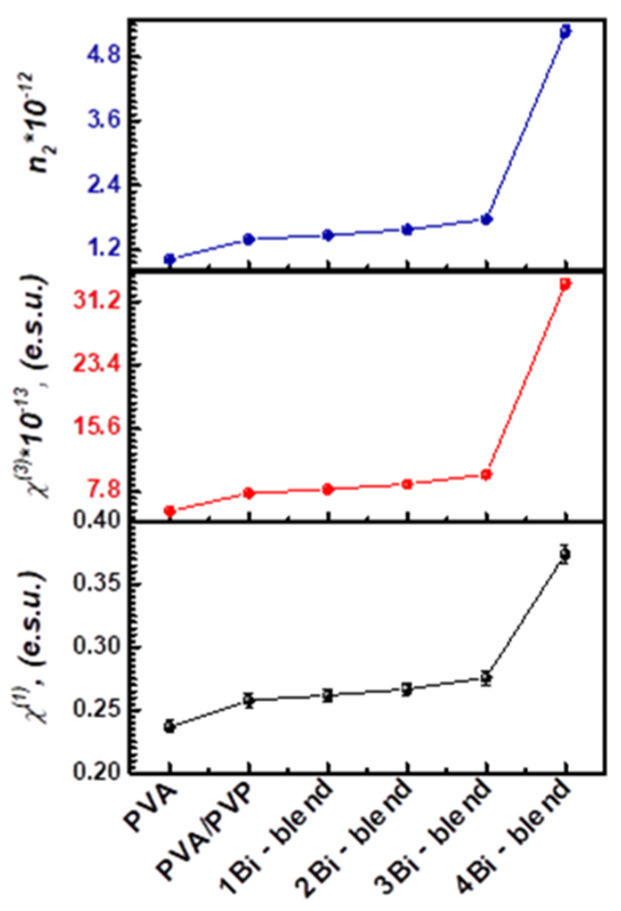
Non-linear optical parameters $\chi^{\left(1\right)}$, $\chi^{\left(3\right)}$, and *n*_2_ for pristine PVA, PVA/PVP blend, and Bi-blend hybrid films.

The first-order $\chi^{\left(1\right)}$ and nonlinear parameters ($\chi^{\left(3\right)}$ and *n*_2_) are summarized in Table 2. The larger values are observed for 3Bi-blend, as shown in Figure 13. At higher Bi-metal concentrations, more particles absorb extra electromagnetic waves, leading to high polarization of the polymer films and an improvement in the nonlinear parameters. This means that as the transition energy gap decreased, the nonlinear properties of the polymeric composite enhanced. The present values of optical susceptibilities and *n*_2_ are higher than those doped with SnS_2_/Fe [49]. Therefore, the as-prepared hybrid films could be used in nonlinear optoelectronic devices.

### 3.5. Optical Limiting Characterization

**Figure 14:**
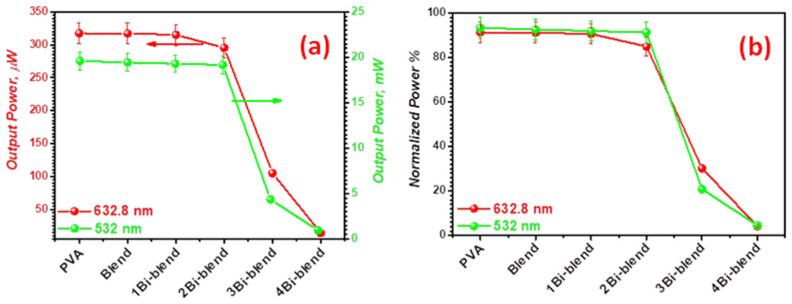
(**a**) Output power, and (**b**) normalized power for Bi-blend hybrid films.

Optical limiters are devices made to filter the incident electromagnetic waves [14]. The protection of optical sensors and components from laser deterioration is one of the most commonly used application fields of this effect [62,63]. Therefore, the output power and normalized (output/input) power of two distinct laser sources (green and He-Ne laser sources) with wavelengths of 533 nm and 632.8 nm, respectively, were operated to investigate the optical limiting characteristics (OLC) for the films under examination. Table 3 summarizes the optical limiting parameters for each source. Figure 14a indicates that the values of the used source’s output powers are large for pristine PVA, blend, and blend: 0–3.7 wt.% Bi polymer films. However, increasing the Bi-metal concentration in the blend matrix to 3.7 wt.% reduces the output power from 317.5 to 14.2 μW and 19.6 to 4.57 mW, respectively, for lasers with 533 nm and 632.8 nm. Hence, the filler concentration plays a significant role in the OLC. The variation in the output power values between the two sources is due to the composite film’s reaction sensitivity to incident light. A sample with greater Bi-metal nanopowder concentrations has more molecules per unit volume in the blend matrix, which participates in the optical interactions during nonlinear absorption mechanisms [14]. Consequently, the OLC of polymer films is correlated with the sample’s ability to absorb and scatter light. As shown in Figure 14b, the 3Bi-blend polymer sample produced the lowest value of normalized power. Therefore, the sample can be used as an optical limiting laser, since the light power is strongly attenuated.

## 4. Conclusions

Facile synthesized PVA/PVP polymeric composite films with different concentrations of Bi-metal were prepared using the low-cost solution casting process. The (101) diffraction peak of PVA is affected by PVP and the Bi-metal’s weight percentage, which indicates a reduction in crystallinity. The morphological surface change was observed via SEM with increasing Bi-metal concentration up to 3.7 wt.%. The optical absorption spectrum in UV–Vis of the blend film was influenced by Bi-metal content in the matrix. The absorption edge shifted from 5.254 eV to 3.8 eV, while the Urbach energy changed from 0.566 eV to 3.007 eV, respectively. Therefore, there is a reduction in the energy required for the electronic transition from VB to CB. The optical bandgap of the films reduced from 5.02 eV for a pristine blend to 3.03 eV for Bi-blend. As a function of the energy bandgap values, the refractive index increased from 1.99 to 2.38. The nonlinear *χ*^(3)^ and *n*_2_ parameters results and OLC of the films indicate that we can conclude that the Bi-embedded blend films are promising materials for waveguides, aircraft windows, laser absorbers, nonlinear optical applications, and optoelectronics.

## Figures and Tables

**Table 2:** Dielectric, L/NL optical parameters of Bi-blend hybrid films.

Films	*n*	$\varepsilon_{\infty }$	$\varepsilon_{o}$	$\chi^{\left(1\right)}\;(e.s.u.)$	$\chi^{\left(3\right)}$ $\times 10^{-13}\;(e.s.u.)$	$n_{2}\;\times 10^{-11}$
PVA	1.996	3.984	263.12	0.237	5.416	1.022
PVA/PVP blend	2.062	4.251	171.45	0.258	7.638	1.395
1Bi-blend	2.074	4.301	158.02	0.262	8.115	1.474
2Bi-blend	2.089	4.363	143.18	0.267	8.747	1.577
3Bi-blend	2.115	4.473	120.03	0.276	9.940	1.771
4Bi-blend	2.388	5.702	16.90	0.374	33.405	5.271
10% SnS_2_/Fe-blend [49]	1.750	5.039	−	0.040	4.1 × 10^−16^	1.25 × 10^−14^

**Table 3:** Optical limiting parameters for Bi-blend hybrid samples.

Films	He–Ne Laser of 632.8 nm I_o_ = 348.9 μW		Green Laser of 533 nm I_o_ = 20.06 mW	
	Output Power (μW) ± 0.05	Normalized Power (%)	Output Power (mW) ± 0.05	Normalized Power (%)
PVA	317.5	91.24	19.60	93.33
PVA/PVP blend	317.2	91.15	19.44	92.57
1Bi-blend	315.2	90.57	19.29	91.86
2Bi-blend	295.5	84.91	19.17	91.28
3Bi-blend	105.1	30.20	4.37	20.81
4Bi-blend	14.2	4.081	0.96	4.57

## Data Availability

The data presented in this study are available on request from the corresponding author.
